# Perioperative chemotherapy with 5-FU, leucovorin, oxaliplatin, and docetaxel (FLOT) for esophagogastric adenocarcinoma: ten years real-life experience from a surgical perspective

**DOI:** 10.1007/s00423-023-02822-7

**Published:** 2023-02-10

**Authors:** Leila Sisic, Nerma Crnovrsanin, Henrik Nienhueser, Jin-On Jung, Sabine Schiefer, Georg Martin Haag, Thomas Bruckner, Martin Schneider, Beat P. Müller-Stich, Markus W. Büchler, Thomas Schmidt

**Affiliations:** 1 Department of Surgery, University Hospital Heidelberg, 69120 Heidelberg, Germany; 2 Present Address: Department of General, Visceral, Cancer and Transplant Surgery, University Hospital of Cologne, 50937 Cologne, Germany; 3 Department of Medical Oncology, National Center for Tumor Diseases (NCT), University Hospital Heidelberg, 69120 Heidelberg, Germany; 4 Institute for Medical Biometry (Imbi), University Hospital Heidelberg, 69120 Heidelberg, Germany

**Keywords:** Adenocarcinoma, Esophageal cancer, Gastric cancer, Histopathological regression, Perioperative chemotherapy

## Abstract

**Purpose:**

According to the results of FLOT4 trial, perioperative FLOT chemotherapy improved overall survival (OS) in locally advanced, resectable esophagogastric adenocarcinoma (EGA) compared to perioperative ECF/ECX. We report real-life data 10 years after introduction of perioperative FLOT at our institution.

**Methods:**

Survival of 356 consecutive EGA patients (cT3/4 and/or cN + and/or cM1) who underwent curative surgical resection was retrospectively analysed from a prospective database. A total of 263 patients received preoperative chemotherapy according to FLOT protocol and 93 patients received an epirubicin/platinum/5FU-based regimen (EPF). Propensity score matching (PSM) according to pretretment characteristics was performed to compensate for heterogeneity between groups.

**Results:**

Median OS did not differ between groups (FLOT/EPF 52.1/46.4 months, *p* = 0.577). After PSM, survival was non-significantly improved after FLOT compared to EPF (median OS not reached/46.4 months, *p* = 0.156). Perioperative morbidity and mortality did not differ between groups. Histopathologic response rate was 35% after FLOT and 26% after EPF (*p* = 0.169). R0 resection could be achieved more frequently after FLOT than after EPF (93%/79%, *p* = 0.023).

**Conclusion:**

Overall survival after perioperative FLOT followed by surgery is comparable to clinical trials. However, collective real-life application of FLOT failed to provide a significant survival benefit compared to EPF. In clinical reality, patient selection is triggered by age, comorbidity, tumor localization, and clinical tumor stage. Yet matched analyses support FLOT4 trial findings.

**Supplementary Information:**

The online version contains supplementary material available at 10.1007/s00423-023-02822-7.

## Introduction

Over the past decades, treatment of esophagogastric adenocarcinoma (EGA) has developed from mere tumor resection to sophisticated multimodal treatment strategies, in order to overcome limitations of surgery alone by improving local resectability as well as systemic tumor control [1–9]. In Western countries, where more than 70% of junctional and gastric adenocarcinomas are diagnosed in advanced stages [10], perioperative chemotherapy has become standard treatment for locally advanced EGA [11, 12] after 2006 based on the results of the MAGIC trial [1]. Hence, perioperative triplet epirubicin-, platinum-, and fluorouracil-based chemotherapy regimens (EPF) became state of the art in treating locally advanced EGA. Results of the MAGIC trial later were confirmed by the French FNCLCC/FFCD trial using a platinum/fluoropyrimidine doublet therapy [2]. Despite these advances, outcome of EGA patients remained unsatisfactory. A new combination consisting of fluorouracil, leucovorin, oxaliplatin, and docetaxel (FLOT) was first evaluated in metastatic EGA and proved to be highly active [13–15]. Thereupon, a phase II/III randomized controlled trial (RCT) was conducted comparing perioperative FLOT to perioperative anthracycline-based triplet chemotherapy with epirubicin, cisplatinum, 5-fluorouracil/capecitabine (ECF/ECX) for treatment of locally advanced EGA. The FLOT4 trial showed a significantly higher complete pathologic response (pCR) rate of 16% after FLOT compared to 6% after ECF/ECX [16] and revealed increased OS in the FLOT group compared to the ECF/ECX group (50 vs. 35 months median) [17]. These results internationally defined FLOT as the new standard perioperative chemotherapy protocol for treatment of EGA. However, data from real-life application of perioperative FLOT chemotherapy in clinical practice is scarce. This is the first study to report real-life experience on perioperative FLOT compared to anthracycline-based triplet chemotherapy (EPF). The aim of this single-center retrospective study was to investigate, whether the results of the FLOT4 trial can be reproduced in a heterogeneous patient population with comparable results in a real-life environment.

## Methods

### Study design and patient population

**Fig. 1:**
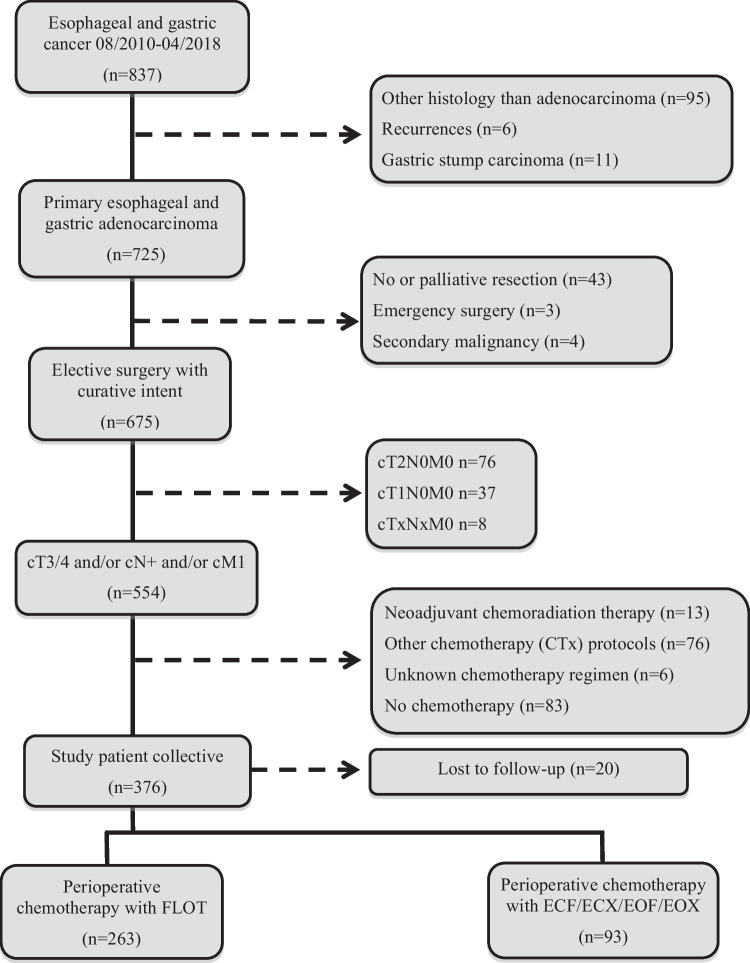
Patient selection and study collective

This study included patients with primary EGA who underwent elective surgery in curative intent at the University Hospital of Heidelberg, Department of Surgery between August 2010 and April 2018. Inclusion criteria were locally advanced primary tumor (cT3/4) and/or nodal positive disease (cN +) and/or distant metastasis (cM1) according to pretherapeutic clinical staging. Patients with distant metastasis (cM1) presented with oligo-metastatic disease and underwent surgery in curative intent as an individual treatment decision as previously described [28]. The metastatic lesions either had complete response to preoperative chemotherapy or were resected additionally to the primary tumor (supplement table). The metastatic lesions were judged to be resectable by experienced surgeons during an interdisciplinary oncological tumor board. All patients received preoperative chemotherapy either according to FLOT (FLOT group) or ECF/ECX/EOF/EOX protocol (EPF group). Clinicopathological and follow-up data of 356 patients (Fig. 1) were collected in a prospective database and analyzed retrospectively.

Informed consent was obtained from all patients. The conduct of this retrospective study was approved by the institutional ethics committee.

### Comorbidity

The American Society of Anaesthesiologists (ASA) Physical Status Classification System was applied in order to assess medical comorbidities and the perioperative risks of patients [18–20]. Pretreatment comorbidities of patients were assessed by experienced anesthesiologists and surgeons. The following conditions, which were clinically judged pertinent to perioperative risk assessment, were considered severe: decompensated renal insufficiency, decompensated cardiac insufficiency, liver cirrhosis, status post (s/p) myocardial infarction, s/p valve replacement, s/p stroke, s/p carotid stenosis, severe coronary heart disease, complicated diabetes mellitus, chronic pancreatitis, chronic obstructive pulmonary disease (COPD), or lung emphysema.

### Pretreatment staging

Initial staging comprised upper endoscopy including biopsies with or without endoscopic ultrasound and computerized tomography (CT) of the chest and upper abdomen for all patients. Clinical tumor stage and localization were assessed according to the 8th edition of the UICC (Union for International Cancer Control) staging system. As for the cN category, it was only differentiated between cN0 and cN + according to lymph node diameter, shape, and contrast enhancement. The cM category was evaluated by CT or biopsies.

### Chemotherapy

Chemotherapy was administered in form of FLOT (*n* = 263), ECF/ECX (*n* = 37), or EOF/EOX protocol (*n* = 46) according to FLOT4, MAGIC, and REAL-2 trials as described previously. Surgery was scheduled 4–6 weeks after the last application of preoperative chemotherapy. Restaging was done before surgery in form of CT of the chest and abdomen and endoscopy if necessary. Adjuvant treatment was administered in form of perioperative treatment if patients had undergone neoadjuvant treatment [21]. Postoperative chemotherapy started 4–8 weeks after surgery. Patients received treatment on an outpatient basis either by the National Center for Tumor Diseases Heidelberg (NCT) or other treating oncologists.

### Surgery

The surgical approach for tumor resection was chosen depending on tumor localization. Standard surgical procedures were a right abdominothoracic en-bloc esophagectomy with a 2-field lymphadenectomy (Ivor-Lewis procedure) for adenocarcinoma of the esophagogastric junction (AEG) I, a transhiatal extended gastrectomy (THG) with an extended D2 lymphadenectomy for AEG III and or proximal gastric cancer (GC), and AEG II were treated either like AEG I or III depending on tumor extension into the esophagus [22]. In the case of GC, a total gastrectomy with D2 lymphadenectomy was performed for tumors in the middle or distal third of the stomach, and a subtotal gastrectomy for distal GC, if an adequate proximal resection margin was possible. All perioperative complications were recorded and graded according to Clavien-Dindo classification [23]. In patients with cM1 and intraoperative confirmation of metastasis, the surgical procedure was extended by resection of the metastatic lesions.

### Histopathology and postoperative staging

Resection specimens were histologically examined in the Department of Pathology of the University Hospital. Histopathologic work up included extent of primary tumor, regional lymph node spread, presence of distant metastases ((y)pTNM categories according to the UICC classification, 8th edition), R category, tumor differentiation, and growth pattern according to Laurén. The lymph node ratio was calculated by dividing the number of LNs involved by the total number of LNs removed [24]. Tumor regression was evaluated according to Becker [25]. Less than 10% residual vital primary tumor (Becker Ia + Ib) was classified as histopathological response.

### Follow-up

Patients were followed on an outpatient basis by our Medical Oncology Department according to a standardized protocol or other treating physicians as previously published [26]. Last follow-up was September 2, 2020. Complete follow-up information was available for all 439 patients. The median follow-up time for surviving patients *(n* = 219) was 60.2 months.

### Propensity score matching

In order to reduce effects of selection bias and confounding factors in survival comparison, propensity score matching (PSM) was performed to create comparable groups. Propensity scores were estimated using a multivariable logistic regression model with the treatment groups (FLOT vs. EPF) as the dependent variables and potential confounders (age, sex, BMI, ASA, severe, cardiovascular, and pulmonary comorbidities, tumor localization, grading, Laurén classification, signet ring cells, cT, cN, and cM category) as covariates. One-to-one matching without replacement was performed using a 0.1 caliper width [27]. This allowed us to generate 81 (FLOT vs. EPF) score-matched pairs, which were used in subsequent analyses as indicated.

### Statistical analysis

Overall survival (OS) was calculated from time of diagnosis till death or last follow-up. Survival rates were estimated using the Kaplan–Meier method. Differences in survival amongst groups were calculated using the log-rank test. To compare categorical variables, we used χ^2^ and Fisher exact test, for comparison of continuous variables the Mann–Whitney-*U* test was used. All tests were two-sided and a *p*-value < 0.05 was considered as statistically significant. Analyses were performed using SPSS version 20.0 (SPSS Inc., Chicago, Illinois, USA).

## Results

### Pretreatment patient and tumor characteristics

The median age at time of diagnosis was 61 (range 25–82) years. 208 patients (58.4%) were diagnosed with junctional (AEG) and 148 (41.6%) with gastric cancer (GC). FLOT chemotherapy was administered to 263 (73.9%) and epirubicin-based triplet chemotherapy (EPF) to 93 (26.1%) patients. Detailed information on patient demographics, tumor characteristics, and treatment is summarized in Table 1.

**Table 1:** a) Pretreatment, b) treatment-associated, and c) postoperative patient and tumor characteristics of all patients

	FLOT (*n* = 263)		EPF (*n* = 93)			*p*-value		Total (*n* = 356)	
a) Pretreatment characteristics									
Age at diagnosis [years]									
Median*	60	(25–82)	61	(27–79)		0.366		60	(25–79)
≤ 45	31	11.8%	13	14.0%		0.549		44	12.4%
46–69	191	72.6%	62	66.7%				253	71.1%
≥ 70	41	15.6%	18	19.4%				59	16.6%
Sex						0.687			
Male	191	72.6%	65	69.9%				256	71.9%
Female	72	27.4%	28	30.1%				100	28.1%
Body mass index [kg/m^2^]						0.410			
Underweight (< 18.5)	5	1.9%	2	2.2%				7	2.0%
Normal weight (18.5–24.9)	120	45.6%	44	47.3%				164	46.1%
Overweight (25–34.9)	134	51.0%	46	49.5%				180	50.6%
Obesity (≥ 35)	0	0.0%	1	1.1%				1	0.3%
Median*	25.5	(15.8–52.1)	26.0	(18.0–36.1)		0.699		25.6	(15.8–36.1)
ASA classification**						0.410			
ASA I	5	1.9%	2	2.2%				7	2.0%
ASA II	120	45.6%	44	47.3%				164	46.1%
ASA III	134	51.0%	46	49.5%				180	50.6%
ASA IV	0	0.0%	1	1.1%				1	0.3%
Comorbidity									
Severe comorbidities	46	17.5%	18	19.4%		0.754		64	17.9%
Cardiovascular	96	36.5%	36	38.7%		0.710		132	37.1%
Pulmonary	32	12.2%	11	11.8%		1.000		43	12.1%
Tumor localization						0.804			
AEG I	60	22.8%	22	23.7%				82	23.0%
AEG II	72	27.4%	24	25.8%				96	26.9%
AEG III	20	7.6%	10	10.8%				30	8.4%
Stomach	111	42.2%	37	39.8%				148	41.6%
Grading**						0.843			
G1	4	1.5%	1	1.1%				5	1.4%
G2	69	26.2%	24	25.8%				93	26.1%
G3	155	58.9%	56	60.2%				211	59.3%
G4	2	0.8%	0	0.0%				2	0.8%
Laurén classification**						0.439			
Intestinal	168	63.9%	58	62.4%				226	63.5%
Diffuse	73	27.8%	31	33.3%				104	29.2%
Mixed type	19	7.2%	4	4.3%				23	7.7%
Signet ring cells**						0.609			
No	176	66.9%	60	64.5%				236	66.3%
Yes	84	31.9%	33	35.5%				117	32.8%
cT category**						0.146			
cT1	2	0.8%	0	0.0%				2	0.6%
cT2	12	4.6%	3	3.2%				15	4.2%
cT3	183	69.6%	77	82.8%				260	73.0%
cT4	61	23.2%	13	14.0%				74	20.8%
cN category**						**0.018**			
cN0	21	8.0%	16	17.2%				37	10.4%
cN +	238	90.5%	77	82.8%				315	88.5%
cM category						**0.004**			
cM0	215	81.8%	87	93.5%				300	84.3%
cM1	48	18.2%	6	6.5%				56	15.7%
b) Treatment associated characteristics									
Neoadjuvant treatment									
Complete (≥ 4 × FLOT or ≥ 3 × EPF)		248	94.3%	84	90.3%		0.228	332	93.3%
Incomplete (≥ 1 cycle)		15	5.7%	9	9.7%			24	6.7%
Number of cycles*		4	(2–13)	3	(1–4)		** < 0.001**	4	(1–13)
Type of surgery							0.707		
Subtotal gastrectomy		24	9.1%	13	14.0%			37	10.4%
Total gastrectomy		74	28.1%	27	29.0%			101	28.4%
Transhiatal extended gastrectomy		72	27.4%	25	26.9%			97	27.2%
Transthoracic esophagectomy		87	33.1%	26	28.0%			113	31.7%
Esophagogastrectomy		6	2.3%	2	2.2%			8	2.2%
Morbidity									
Surgical complications		88	33.5%	37	39.8%		0.312	125	35.1%
Clavien Dindo I + II		24	9.1%	11	11.8%		1.000	35	9.8%
Clavien Dindo III–V		64	24.3%	28	30.1%			92	25.8%
Anastomotic leakage		32	12.2%	14	15.1%		0.476	46	12.9%
Medical complications		61	23.2%	31	33.3%		0.073	92	25.8%
Cardiac		19	7.2%	11	11.8%		0.193	30	8.4%
Pulmonary		44	16.7%	23	24.7%		0.093	67	18.8%
ICU stay [days]*		3	(0–92)	3	(0–71)		0.381	3	(0–92)
Hospital stay [days]*		15	(1–126)	16	(8–127)		0.438	15	(1–127)
Mortality									
30-day mortality		2	0.8%	2	2.2%		0.280	4	1.2%
In-hospital mortality		4	1.5%	5	5.4%		0.056	9	2.5%
Adjuvant treatment**									
None		69	26.2%	30	32.3%		0.345	99	27.8%
Yes		184	70.0%	61	65.6%			245	68.8%
Chemoradiotherapy		11	4.2%	7	7.5%		0.163	18	7.3%
Chemotherapy		173	65.8%	54	58.1%			227	92.7%
FLOT		138	52.5%	3	3.2%		** < 0.001**	141	62.1%
FLO		27	10.3%	1	1.1%			28	12.3%
EPF		1	0.4%	46	49.5%			47	20.7%
Other		7	2.7%	3	3.2%			10	4.4%
c) Postoperative patient and tumor characteristics									
(y)pT category							0.061		
(y)pT0		17	6.5%	11	11.8%			28	7.9%
(y)pT1 (a + b)		33	12.5%	4	4.3%			37	10.4%
(y)pT2		34	12.9%	17	18.3%			51	14.3%
(y)pT3		129	49.0%	47	50.5%			176	49.4%
(y)pT4 (a + b)		50	19.0%	14	15.1%			64	18.0%
(y)pN category							0.064		
(y)pN0		110	41.8%	44	47.3%			154	43.2%
(y)pN1		43	16.3%	7	7.5%			50	14.0%
(y)pN2		39	14.8%	21	22.6%			60	16.8%
(y)pN3		71	27.0%	21	22.6%			92	25.8%
Lymph nodes									
Number of positive lymph nodes*		1	(0–44)	1	(0–36)		0.409	2	(0–44)
Number of lymph nodes removed*		29	(8–93)	27	(2–59)		0.151	28	(2–93)
Lymph node ratio*		0.05	(0–0.96)	0.05	(0–0.88)		0.493	0.05	(0–0.96)
0.0		110	41.8%	44	47.3%		0.571	154	43.3%
< 0.2		62	23.6%	22	23.7%			84	23.6%
≥ 0.2		91	34.6%	27	29.0%			118	33.1%
(y)pM category							0.222		
(y)pM0		223	84.8%	84	90.3%			307	86.2%
(y)pM1		40	15.2%	9	9.7%			49	13.8%
R category									
Locally R0		229	87.1%	75	80.6%		0.171	304	85.4%
Locally Rx/R1/R2		34	12.9%	18	19.4%			52	14.6%
Metastases R0		26	9.9%	3	3.2%		0.133	29	8.1%
Metastases Rx/R1/R2		14	5.3%	6	6.5%			20	5.6%
All lesions R0		220	83.7%	73	78.5%		0.271	293	82.3%
All lesions Rx/R1/R2		43	16.3%	20	21.5%			63	17.7%
Histopathologic regression**									
Response (Ia + Ib)		84	31.9%	25	26.9%		0.297	109	30.6%
Non-response (II + III)		168	63.9%	68	73.1%			236	66.3%
Becker grade Ia		16	6.1%	11	11.8%		0.059	27	7.6%
Becker grade Ib		69	26.2%	14	15.1%			83	23.3%
Becker grade II		77	29.3%	30	32.3%			107	30.1%
Becker grade III		90	34.2%	38	40.9%			128	36.0%

^*^Median (range)

^**^Data was not available for all patients; *NA* not applicable; values in bold print indicate a significance-level of *p* ≤ 0.05

In comparison to the EPF group, more patients were clinically staged cN + (90.5% vs. 82.8%,* p* = 0.018) and cM1 (18.2 vs. 6.5%, *p* = 0.004) in the FLOT group (Table 1a). To create comparable groups, a propensity score matching (PSM) was performed. Comparison of pretreatment characteristics after PSM between 81 FLOT vs. 81 EPF patients revealed no significant differences (Table 2a).

**Table 2:** a) Pretreatment, b) treatment-associated, and c) postoperative patient and tumor characteristics of matched cohorts

	FLOT (*n* = 81)			EPF (*n* = 81)			*p*-value
a) Pretreatment characteristics							
Age at diagnosis [years]							
Median*	58	(31–75)		61		(27–79)	0.410
≤ 45	7	8.6%		12		14.8%	0.330
46–69	63	77.8%		55		67.9%	
≥ 70	11	13.6%		14		17.3%	
Sex							1.000
Male	54	66.7%		54		66.7%	
Female	27	33.3%		27		33.3%	
Body mass index [kg/m^2^]							
Underweight (< 18.5)	2	2.5%		2		2.5%	0.936
Normal weight (18.5–24.9)	38	46.9%		34		42.0%	
Overweight (25–34.9)	25	30.9%		28		34.6%	
Obesity (≥ 35)	16	19.8%		17		21.0%	
Median*	25.3	(16.7–43.8)		25.8		(18.0–39.1)	0.828
ASA classification**							0.340
ASA I	0	0.0%		2		2.5%	
ASA II	44	54.3%		39		48.1%	
ASA III	37	45.7%		39		48.1%	
ASA IV	0	0.0%		1		1.2%	
Comorbidity							
Severe comorbidities	16	19.8%		14		17.3%	0.840
Cardiovascular	33	40.7%		29		35.8%	0.628
Pulmonary	9	11.1%		11		13.6%	0.812
Tumor localization							0.868
AEG I	22	27.2%		20		24.7%	
AEG II	20	24.7%		19		23.5%	
AEG III	6	7.4%		9		11.1%	
Stomach	33	40.7%		33		40.7%	
Grading**							0.480
G1	2	2.5%		1		1.2%	
G2	30	37.0%		24		29.6%	
G3	49	60.5%		56		69.1%	
G4	0	0.0%		0		0.0%	
Laurén classification**							0.260
Intestinal	57	70.4%		47		58.0%	
Diffuse	21	25.9%		30		37.0%	
Mixed type	3	3.7%		4		4.9%	
Signet ring cells**							0.184
No	58	71.6%		49		60.5%	
Yes	23	28.4%		32		39.5%	
cT category**							0.337
cT1	2	2.5%		0		0.0%	
cT2	3	3.7%		3		3.7%	
cT3	61	75.3%		68		84.0%	
cT4	15	18.5%		10		12.3%	
cN category**							0.521
cN0	11	13.6%		15		18.5%	
cN +	70	86.4%		66		81.5%	
cM category							1.000
cM0	76	93.8%		75		92.6%	
cM1	5	6.2%		6		7.4%	
b) Treatment-associated characteristics							
Neoadjuvant treatment							
Complete (≥ 4 × FLOT or ≥ 3 × EPF)		75	92.6%		72	88.9%	0.589
Incomplete (≥ 1 cycle)		6	7.4%		9	11.1%	
Number of cycles*		4	(2–6)		3	(1–4)	** < 0.001**
Type of surgery							0.616
Subtotal gastrectomy		6	7.4%		11	13.6%	
Total gastrectomy		23	28.4%		24	29.6%	
Transhiatal extended gastrectomy		23	28.4%		21	25.9%	
Transthoracic esophagectomy		26	32.1%		24	29.6%	
Esophagogastrectomy		3	3.7%		1	1.2%	
Morbidity							
Surgical complications		32	39.5%		31	38.3%	1.000
Clavien Dindo I + II		8	9.9%		8	9.9%	1.000
Clavien Dindo III–V		24	29.6%		23	28.4%	
Anastomotic leakage		14	17.3%		12	14.8%	0.831
Medical complications		19	23.5%		23	28.4%	0.591
Cardiac		5	6.2%		7	8.6%	0.766
Pulmonary		14	17.3%		19	23.5%	0.436
ICU stay [days]*		3	(0–92)		3	(0–71)	0.547
Hospital stay [days]*		16	(7–112)		16	(8–86)	0.917
Mortality							
30-day mortality		0	0.0%		1	1.2%	1.000
In-hospital mortality		1	1.2%		3	3.7%	0.620
Adjuvant treatment**							
None		25	30.9%		25	30.9%	1.000
Yes		52	64.2%		55	67.9%	
Chemoradiotherapy		2	2.5%		6	7.4%	0.272
Chemotherapy		50	61.7%		49	60.5%	
FLOT		41	50.6%		3	3.7%	** < 0.001**
FLO		8	9.9%		1	1.2%	
EPF		0	0.0%		41	50.6%	
Other		1	1.2%		3	3.7%	
c) Postoperative patient and tumor characteristics							
(y)pT category							**0.045**
(y)pT0		4	4.9%		10	12.3%	
(y)pT1 (a + b)		15	18.5%		4	4.9%	
(y)pT2		11	13.6%		15	18.5%	
(y)pT3		41	50.6%		40	49.4%	
(y)pT4 (a + b)		10	12.3%		12	14.8%	
(y)pN category							0.317
(y)pN0		36	44.4%		38	46.9%	
(y)pN1		11	13.6%		5	6.2%	
(y)pN2		13	16.0%		19	23.5%	
(y)pN3		21	25.9%		19	23.5%	
Lymph nodes							
Number of positive lymph nodes*		2	(0–44)		1	(0–36)	0.669
Number of lymph nodes removed*		28	(8–50)		25	(2–54)	0.584
Lymph node ratio*		0.06	(0–0.96)		0.05	(0–0.88)	0.695
0.0		36	44.4%		38	46.9%	0.791
< 0.2		16	19.8%		18	22.2%	
≥ 0.2		29	35.8%		25	30.9%	
(y)pM category							0.781
(y)pM0		75	92.6%		73	90.1%	
(y)pM1		6	7.4%		8	9.9%	
R category							**0.023**
Locally R0		75	92.6%		64	79.0%	
Locally Rx/R1/R2		6	7.4%		17	21.0%	
Metastases R0		6	7.4%		2	2.5%	**0.010**
Metastases Rx/R1/R2		0	0.0%		6	7.4%	
All lesions R0		75	92.6%		62	76.5%	**0.008**
All lesions Rx/R1/R2		6	7.4%		19	23.5%	
Histopathologic regression**							
Response (Ia + Ib)		28	34.6%		21	25.9%	0.169
Non-response (II + III)		48	59.3%		60	74.1%	
Becker grade Ia		4	4.9%		10	12.3%	**0.024**
Becker grade Ib		24	29.6%		11	13.6%	
Becker grade II		26	32.1%		27	33.3%	
Becker grade III		22	27.2%		33	40.7%	

^*^Median (range)

^**^Data was not available for all patients; *NA* not applicable; values in bold print indicate a significance-level of *p* ≤ 0.05

### Treatment

All planned cycles of preoperative chemotherapy were completed by 94.3% of patients in the FLOT group and 90.3% in the EPF group (*p* = 0.228). Preoperatively a median of 4 (2–13) cycles of FLOT and 3 (1–4) cycles of EPF were given. An overview on chemotherapy and surgical treatment is given in Table 1b and Table 2b.

### Perioperative morbidity

Overall surgical complication rate was 35.1%. Of all surgical complications 25.8% were grade 3 or higher according to Clavien-Dindo. Anastomotic leakage occurred in 12.9% of patients. 30-day mortality was 1.2% and in-hospital mortality was 2.5%. While FLOT and EPF groups differed little regarding surgical complications, there was a trend towards more medical and especially more pulmonary complications in EPF compared to FLOT patients (Table 1b and Table 2b).

### Histopathology

Distribution of individual (y)pT categories was distinct between FLOT and EPF groups (Table 1c and Table 2c). Yet the proportion of locally advanced primary tumors ((y)pT3/4) was similar in the FLOT and EPF groups before (68.1% vs. 65.6%, *p* = 0.700) and after PSM (63.0% vs. 64.2%, *p* = 1.000).

A median of 28 lymph nodes (LNs) was removed and a median of 1 lymph node was positive, resulting in a median lymph node ratio (LNR) of 0.05. Distribution of individual (y)pN categories did not differ significantly (Table 1c). After PSM, the median number of positive and removed lymph nodes, and the distribution of LNR and ypN categories was similar (Table 2c).

The FLOT group revealed a trend towards higher R0 resection rates than the EPF group and after PSM this difference proved to be significant (R0 92.6 vs. 79.0%, *p* = 0.023) (Table 1c and Table 2c).

There was a trend towards a higher histopathologic response rate after FLOT compared to EPF before (31.9 vs. 26.9%, *p* = 0.297) and after PSM (34.6 vs. 25.9%, *p* = 0.169). However, the pCR rate (Becker Ia) was higher in the EPF group (6.1 vs. 11.8%, *p* = 0.059, after PSM 4.9 vs. 12.3%, *p* = 0.024), whereas after FLOT a markedly greater proportion of patients revealed regression grade Becker Ib (26.2 vs. 15.1%, *p* = 0.059, after PSM 29.6 vs. 13.6%, *p* = 0.024) (Table 1c and Table 2c).

### Survival

Upon last follow-up, 183 of 356 patients (51.4%) had died (FLOT *n* = 130, EPF *n* = 53). Median OS from time of diagnosis for all patients was 49.7 (95% CI 28.3–71.1) months. Estimated 3- and 5-year OS rate was 55.8% and 46.6%.

**Fig. 2:**
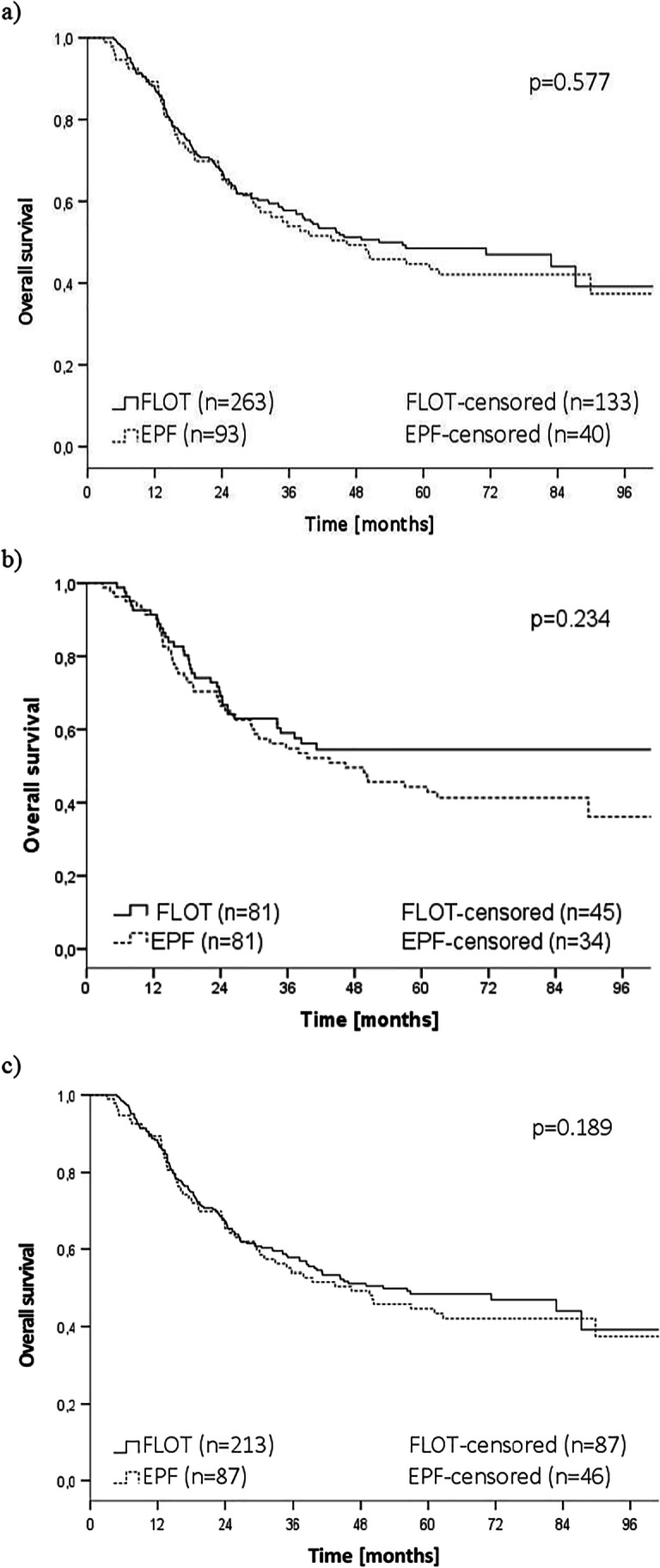
Overall survival according to treatment strategy (perioperative FLOT- vs. epirubicin-based chemotherapy (EPF)) in **a** all patients, **b** matched cohorts, **c** patients without distant metastases (cM0)

Median OS was 52.1 (95% CI 27.4–76.8) months for FLOT and 46.4 (95% CI 24.3–68.5) months for EPF (Fig. 2a). Estimated 3- and 5-year OS rate was 57.8% and 48.5% respectively in the FLOT group, 53.9% and 44.7% in the EPF group. There was no significant difference in OS between FLOT and EPF groups (*p* = 0.577).

After PSM, there was a trend towards improved OS after FLOT compared to EPF (median OS not reached/46.4 months, 3-year OS 59.0/54.8%, 5-year OS 54.5/44.3%, *p* = 0.234) (Fig. 2b).

There was also no significant difference in OS in the subgroup analysis of patients without distant metastasis (cM0) with 87.2 months median (95% CI 43.9–130.5) after FLOT vs. 50.2 months median (95% CI 13.7–86.8) after EPF chemotherapy (*p* = 0.189) (Fig. 2c).

### Subgroup analyses

**Fig. 3:**
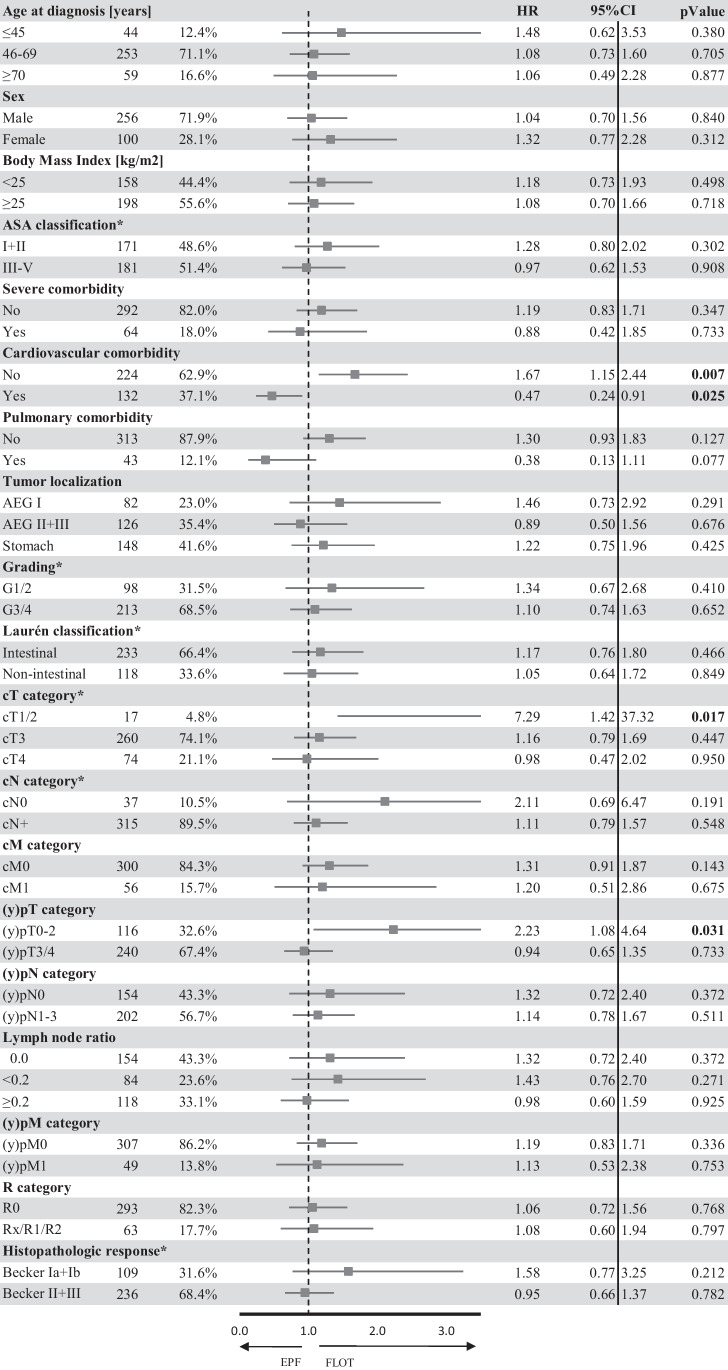
Treatment effect of FLOT compared to EPF on overall survival according to pretreatment and postoperative patient and tumor characteristics. The forest plot shows hazard ratios for death (oblongs) and 95% confidence intervals (I bars). *Data was not available for all patients; values in bold print indicate a significance-level of *p* ≤ 0.5

The relative treatment effect of FLOT on OS was compared to EPF (Fig. 3) across subgroups according to pretreatment and postoperative characteristics. A significant OS benefit in the FLOT group compared to the EPF group could be detected in patients without cardiovascular comorbidities (*p* = 0.007) and locally limited primary tumors in clinical (cT1/2; *p* = 0.006) and pathological (ypT0/1/2; *p* = 0.027) staging. No significant OS difference could be detected in the remaining subgroups.

### Patients with oligo-metastatic disease

In the study, we included 54 patients who presented with distant metastases in the clinical staging at time of diagnosis. A total of 48 of those patients received FLOT and 6 EPF. After the chemotherapy treatment, 31 (64.4%) patients in the FLOT group and 4 (66.7%) patients in the EPF group still presented with oligo-metastatic disease in preoperative restaging investigations. Furthermore, some of the metastatic lesions had complete response to preoperative chemotherapy and could not be detected during the surgery or could not be confirmed by the pathological examination neither during the surgery by frozen section nor the final pathological examination. An overview of the exact localization of the metastasis is given in the supplement table.

## Discussion

The randomized controlled FLOT4 trial demonstrated improved OS in patients with EGA treated with perioperative FLOT chemotherapy compared to ECF/ECX. Median OS was increased by 15 months (50 vs. 35 months) and the estimated 5-year OS rate by 9% (45% vs. 36%) [17]. In our retrospective single-center study, we analyzed, real-life data on survival after perioperative FLOT chemotherapy compared to perioperative epirubin, platinum, and fluorouracil-based triplet chemotherapy 10 years after introduction of FLOT protocol at our institution. Our data show a median OS of 52 months (5-year OS rate 49%) after perioperative FLOT and surgery which is comparable to a median OS of 50 months (5-year OS rate 45%) in the FLOT4 trial [17]. However, in our series, patients who failed to proceed to surgery or did not undergo resection in curative intent were excluded from our study collective, whereas data from the FLOT-4 trial resulted from an intent-to-treat analysis with 5% of all patients who were not resected, mostly due to disease progression. On the other hand, we included selected patients with metastatic disease (18.2% with cM1 and 15% with pM1 in the FLOT group), who nevertheless underwent resection in curative intent as individual treatment decision as previously described [28]. Our results demonstrate a slightly worse survival than an analysis of Glatz et al. with 228 EGA patients all treated with perioperative FLOT followed by curative resection reporting a median OS of 61 months (5-year OS rate 51%) and a median recurrence free survival (RFS) of 42 months (5-year RFS rate 46%) [29]. However, this study included only patients after surgical resection without metastasis. In survival analyses of the matched FLOT cohorts with less advanced cT and cN categories, and especially a lesser proportion of metastatic disease, median OS was excellent (not reached/87 months, 5-year OS rate 55%/62% respectively).

Overall survival of the EPF group in our study was longer (46 months median, 5-year OS rate 45%) than in the ECF/ECX arm of the FLOT4 trial (35 months median, 5-year OS rate 36%) and the ECF arm of the MAGIC trail (25 months median, 5-year OS rate 36%) [1, 17]. This could be explained by the fact that in contrast to the FLOT4 or MAGIC trial we included only patients, who underwent surgery in curative intent.

In our collective real-life application of FLOT, chemotherapy failed to provide a significant survival benefit compared to EPF. This fact is most likely explained by the patient selection obviously occurring in clinical reality, since FLOT is expected to be the more aggressive treatment associated with more efficacy but also potentially causing more side effects. Patients receiving perioperative FLOT revealed more nodal positive and metastatic disease in clinical staging compared to EPF patients. The presence of nodal and distant metastases represents two of the most important and well-known negative prognosticators in EGA [36–38]. The inclusion of M1 patients might be controversial. However, we did not exclude patients with resectable metastatic disease, in order to reflect clinical reality from a surgical point of view. We rather performed subgroup analyses, which could not reveal a significant survival advantage for FLOT compared to EPF in non-metastatic EGA, neither.

After compensating for heterogeneity between groups by performance of PSM, no significant differences could be detected and preoperative patient and tumor characteristics were mostly well balanced between groups. As a result, survival analyses of the matched cohorts revealed a trend towards improved OS after FLOT compared to EPF. Possibly, these differences in OS failed to reach statistical significance, because patient numbers were limited after PSM.

No significant difference was found between FLOT and EPF groups with regard to pathological staging. Hence, in contrast to the FLOT4 study results, our analyses do not clearly indicate that perioperative FLOT is able to achieve better downstaging than EPF regimens [16, 17]. However, the proportion of histopathological responders (Becker grade Ia + b) was 35% after FLOT and 26% after EPF. Phase II results of the FLOT4 trial yielded comparable histopathological response rates (Becker Ia + b 37% after FLOT vs. 23% after ECF/ECX, *p* = 0.02) [16]. Remarkably, the pCR rate (Becker Ia) in our collective was only 5% in the FLOT compared to 12% in the EPF group contradictory to the results of FLOT4 trial with 16% pCR after FLOT compared to 6% after ECF/ECX [16] and quite low compared to other studies reporting a pCR rate of 14–17% after FLOT chemotherapy and even up to 31% in case of intestinal and/or AEG tumors [29, 39].

In accordance with the results of the FLOT4 trial, perioperative complication and mortality rates as well as duration of ICU and hospital stay were similar in both groups [17]. This is in line with the results of multiple studies including the benchmark RCTs, which have shown no increase in perioperative morbidity and mortality [1–3, 17, 40].

Limitations of the study are the retrospective, non-randomized, and single center design. There was strong selection bias resulting in marked heterogeneity between the FLOT and EPF groups rendering comparison of prognosis between the groups impossible. However, this patient selection reflects clinical reality. Even though propensity score matching was performed, remnant heterogeneity between groups cannot be excluded. Besides, despite a considerable sample size of the entire study population, it might still be too small for adequate subgroup analyses. Low patient numbers in the EPF group resulted in limited sample size of matched cohorts.

Ultimately, the issue of the ideal treatment strategy for EGA remains unresolved. Perioperative FLOT chemotherapy has become standard treatment for patients with locally advanced EGA after results of the FLOT4 trial [17]. Junctional cancers are alternatively treated with preoperative chemoradiation according to the CROSS protocol [3] depending on regional standards. Whether patients with AEG should receive perioperative FLOT or neoadjuvant chemoradiation according to CROSS protocol is currently investigated by the phase III ESOPEC trial [41]. To what extent patients could benefit from the addition of radiation to perioperative chemotherapy in the treatment of resectable EGA is currently evaluated in two RCTs, the RACE [42] and TOPGEAR trials [43, 44]. Further trials are necessary to clarify which treatment strategy is most suitable for particular patient subgroups or even individual patients with EGA. In the recently published CheckMate 577, a global RCT involving patients with resected esophageal or junctional cancer after neoadjuvant chemoradiotherapy, disease-free survival was significantly improved after a 1-year course of adjuvant nivolumab therapy compared to placebo (22.4 vs. 11.0 months median, HR 0.69) [45]. Even though OS data are not mature, adjuvant nivolumab will probably become a new standard of care. The adjuvant use of checkpoint inhibitors in patients undergoing perioperative chemotherapy (KEYNOTE-585, NCT03221426) [46] or definitive chemoradiotherapy (KEYNOTE-975, NCT04210115) [47] is subject of ongoing investigations. Implementation of targeted treatment strategies, such as biomarker-based therapy or immunotherapy with monoclonal antibodies such as trastuzumab, ramucirumab, or checkpoint inhibitors into the perioperative setting, might help to further improve the survival of EGA patients.

## Conclusion

Ten years after the introduction of perioperative FLOT chemotherapy for the treatment of EGA at our institution real-life data revealed an OS of patients undergoing perioperative FLOT followed by surgical resection comparable to clinical trials. However, in our collective, we could not demonstrate a significant survival benefit after perioperative FLOT in comparison to perioperative ECX/ECF/EOX/EOF. Therefore, the current monopoly of FLOT as perioperative chemotherapy regimen of choice for resectable EGA must be critically questioned. Yet analyses of matched cohorts support the findings of the FLOT4 trial. Therefore, currently, FLOT represents the preferred perioperative chemotherapy regimen in the standard treatment of EGA.

### Supplementary Information

Below is the link to the electronic supplementary material.

Supplementary file1 (DOCX 19 KB)

## Data Availability

The data can be made available upon reasonable request to the principal investigator(s).

## References

[1] Cunningham D, Allum WH, Stenning SP (2006). Perioperative chemotherapy versus surgery alone for resectable gastroesophageal cancer. N Engl J Med.

[2] Ychou M, Boige V, Pignon JP (2011). Perioperative chemotherapy compared with surgery alone for resectable gastroesophageal adenocarcinoma: an FNCLCC and FFCD multicenter phase III trial. J Clin Oncol.

[3] van Hagen P, Hulshof MC, van Lanschot JJ (2012). Preoperative chemoradiotherapy for esophageal or junctional cancer. N Engl J Med.

[4] Shapiro J, van Lanschot JJ, Hulshof MC (2015). Neoadjuvant chemoradiotherapy plus surgery versus surgery alone for oesophageal or junctional cancer (CROSS): long-term results of a randomised controlled trial. Lancet Oncol.

[5] Noh SH, Park SR, Yang HK (2014). Adjuvant capecitabine plus oxaliplatin for gastric cancer after D2 gastrectomy (CLASSIC): 5-year follow-up of an open-label, randomised phase 3 trial. Lancet Oncol.

[6] Sasako M, Sakuramoto S, Katai H (2011). Five-year outcomes of a randomized phase III trial comparing adjuvant chemotherapy with S-1 versus surgery alone in stage II or III gastric cancer. J Clin Oncol.

[7] Smalley SR, Benedetti JK, Haller DG (2012). Updated analysis of SWOG-directed intergroup study 0116: a phase III trial of adjuvant radiochemotherapy versus observation after curative gastric cancer resection. J Clin Oncol.

[8] Park SH, Sohn TS, Lee J (2015). Phase III trial to compare adjuvant chemotherapy with capecitabine and cisplatin versus concurrent chemoradiotherapy in gastric cancer: final report of the adjuvant chemoradiotherapy in stomach tumors trial, including survival and subset Analyses. J Clin Oncol.

[9] Park SH, Lim DH, Sohn TS (2021). A randomized phase III trial comparing adjuvant single-agent S1, S-1 with oxaliplatin, and postoperative chemoradiation with S-1 and oxaliplatin in patients with node-positive gastric cancer after D2 resection: the ARTIST 2 trial. Ann Oncol.

[10] Wilke H, Lordick F, Meyer HJ, Stahl M (2013). (Neo)-adjuvant chemo(-radio) therapy for adenocarcinomas of the gastroesophageal junction and the stomach in the West. Dig Surg.

[11] Smyth EC, Verheij M, Allum W (2016). Gastric cancer: ESMO Clinical Practice Guidelines for diagnosis, treatment and follow-up. Ann Oncol.

[12] Moehler M, Al-Batran SE, Andus T (2019). Z Gastroenterol.

[13] Al-Batran SE, Hartmann JT, Hofheinz R (2008). Biweekly fluorouracil, leucovorin, oxaliplatin, and docetaxel (FLOT) for patients with metastatic adenocarcinoma of the stomach or esophagogastric junction: a phase II trial of the Arbeitsgemeinschaft Internistische Onkologie. Ann Oncol.

[14] Al-Batran SE, Pauligk C, Homann N (2013). The feasibility of triple-drug chemotherapy combination in older adult patients with oesophagogastric cancer: a randomised trial of the Arbeitsgemeinschaft Internistische Onkologie (FLOT65+). Eur J Cancer.

[15] Lorenzen S, Pauligk C, Homann N, Schmalenberg H, Jager E, Al-Batran SE (2013). Feasibility of perioperative chemotherapy with infusional 5-FU, leucovorin, and oxaliplatin with (FLOT) or without (FLO) docetaxel in elderly patients with locally advanced esophagogastric cancer. Br J Cancer.

[16] Al-Batran SE, Hofheinz RD, Pauligk C (2016). Histopathological regression after neoadjuvant docetaxel, oxaliplatin, fluorouracil, and leucovorin versus epirubicin, cisplatin, and fluorouracil or capecitabine in patients with resectable gastric or gastro-oesophageal junction adenocarcinoma (FLOT4-AIO): results from the phase 2 part of a multicentre, open-label, randomised phase 2/3 trial. Lancet Oncol.

[17] Al-Batran SE, Homann N, Pauligk C (2019). Perioperative chemotherapy with fluorouracil plus leucovorin, oxaliplatin, and docetaxel versus fluorouracil or capecitabine plus cisplatin and epirubicin for locally advanced, resectable gastric or gastro-oesophageal junction adenocarcinoma (FLOT4): a randomised, phase 2/3 trial. Lancet.

[18] Abouleish AE, Leib ML, Cohen NH (2015). ASA provides examples to Each ASA physical status class. ASA Newsl.

[19] Hurwitz EE, Simon M, Vinta SR (2017). Adding examples to the ASA-physical status classification improves correct assignment to patients. Anesthesiology.

[20] Mayhew D, Mendonca V, Murthy BVS (2019). A review of ASA physical status - historical perspectives and modern developments. Anaesthesia.

[21] Sisic L, Blank S, Nienhüser H et al (2020) The postoperative part of perioperative chemotherapy fails to provide a survival benefit in completely resected esophagogastric adenocarcinoma. Surg Oncol 33:177–188. 10.1016/j.suronc.2017.06.001 10.1016/j.suronc.2017.06.001 28684226

[22] Blank S, Schmidt T, Heger P, et al (2018) Surgical strategies in true adenocarcinoma of the esophagogastric junction (AEG II): thoracoabdominal or abdominal approach? Gastric Cancer 21(2):303–314. 10.1007/s10120-017-0746-1 10.1007/s10120-017-0746-1 28685209

[23] Dindo D, Demartines N, Clavien PA (2004). Classification of surgical complications: a new proposal with evaluation in a cohort of 6336 patients and results of a survey. Ann Surg.

[24] Sisic L, Blank S, Weichert W (2013). Prognostic impact of lymph node involvement and the extent of lymphadenectomy (LAD) in adenocarcinoma of the esophagogastric junction (AEG). Langenbeck’s archives of surgery / Deutsche Gesellschaft fur Chirurgie.

[25] Becker K, Langer R, Reim D (2011). Significance of histopathological tumor regression after neoadjuvant chemotherapy in gastric adenocarcinomas: a summary of 480 cases. Ann Surg.

[26] Sisic L, Strowitzki MJ, Blank S et al (2018) Postoperative follow-up programs improve survival in curatively resected gastric and junctional cancer patients: a propensity score matched analysis. Gastric Cancer 21(3):552–568. 10.1007/s10120-017-0751-4 10.1007/s10120-017-0751-4 28741059

[27] D’Agostino RB (1998). Propensity score methods for bias reduction in the comparison of a treatment to a non-randomized control group. Stat Med.

[28] Schmidt T, Alldinger I, Blank S (2015). Surgery in oesophago-gastric cancer with metastatic disease: treatment, prognosis and preoperative patient selection. Eur j surgical oncology : j Eur Soc Surg Oncol Br Assoc Surg Oncol.

[29] Glatz T, Verst R, Kuvendjiska J et al (2020) Pattern of Recurrence and Patient Survival after Perioperative Chemotherapy with 5-FU, Leucovorin, Oxaliplatin and Docetaxel (FLOT) for Locally Advanced Esophagogastric Adenocarcinoma in Patients Treated Outside Clinical Trials. J Clin Med 9(8):2654. 10.3390/jcm9082654 10.3390/jcm9082654 PMC7464040 32824326

[30] Rice TW, Apperson-Hansen C, DiPaola LM (2016). Worldwide esophageal cancer collaboration: clinical staging data. Dis Esophagus.

[31] Siegel RL, Miller KD, Jemal A (2020). Cancer statistics. CA: cancer j clin.

[32] Park SR, Kim MJ, Ryu KW (2010). Prognostic value of preoperative clinical staging assessed by computed tomography in resectable gastric cancer patients: a viewpoint in the era of preoperative treatment. Ann Surg.

[33] Blank S, Blaker H, Schaible A (2012). Impact of pretherapeutic routine clinical staging for the individualization of treatment in gastric cancer patients. Langenbeck's archives of surgery / Deutsche Gesellschaft fur Chirurgie.

[34] Pacelli F, Papa V, Caprino P, Sgadari A, Bossola M, Doglietto GB (2001). Proximal compared with distal gastric cancer: multivariate analysis of prognostic factors. Am Surg.

[35] Yu X, Hu F, Li C, Yao Q, Zhang H, Xue Y (2018). Clinicopathologic characteristics and prognosis of proximal and distal gastric cancer. Onco Targets Ther.

[36] Rice TW, Chen LQ, Hofstetter WL (2016). Worldwide esophageal cancer collaboration: pathologic staging data. Dis Esophagus.

[37] Rice TW, Lerut TE, Orringer MB (2016). Worldwide Esophageal Cancer Collaboration: neoadjuvant pathologic staging data. Dis Esophagus.

[38] Sano T, Coit DG, Kim HH (2017). Proposal of a new stage grouping of gastric cancer for TNM classification: International Gastric Cancer Association staging project. Gastric cancer : off j Int Gastric Cancer Assoc Jpn Gastric Cancer Assoc.

[39] Homann N, Pauligk C, Luley K (2012). Pathological complete remission in patients with oesophagogastric cancer receiving preoperative 5-fluorouracil, oxaliplatin and docetaxel. Int J Cancer.

[40] Springfeld C, Wiecha C, Kunzmann R (2015). Influence of different neoadjuvant chemotherapy regimens on response, prognosis, and complication rate in patients with esophagogastric adenocarcinoma. Ann Surg Oncol.

[41] Hoeppner J, Lordick F, Brunner T (2016). ESOPEC: prospective randomized controlled multicenter phase III trial comparing perioperative chemotherapy (FLOT protocol) to neoadjuvant chemoradiation (CROSS protocol) in patients with adenocarcinoma of the esophagus (NCT02509286). BMC Cancer.

[42] Lorenzen S, Biederstadt A, Ronellenfitsch U (2020). RACE-trial: neoadjuvant radiochemotherapy versus chemotherapy for patients with locally advanced, potentially resectable adenocarcinoma of the gastroesophageal junction - a randomized phase III joint study of the AIO, ARO and DGAV. BMC Cancer.

[43] Leong T, Smithers BM, Haustermans K (2017). TOPGEAR: A randomized, phase III trial of perioperative ecf chemotherapy with or without preoperative chemoradiation for resectable gastric cancer: interim results from an International, Intergroup Trial of the AGITG, TROG, EORTC and CCTG. Ann Surg Oncol.

[44] Leong T, Smithers BM, Michael M (2015). TOPGEAR: a randomised phase III trial of perioperative ECF chemotherapy versus preoperative chemoradiation plus perioperative ECF chemotherapy for resectable gastric cancer (an international, intergroup trial of the AGITG/TROG/EORTC/NCIC CTG). BMC Cancer.

[45] Kelly RJ, Ajani JA, Kuzdzal J (2021). Adjuvant nivolumab in resected esophageal or gastroesophageal junction cancer. N Engl J Med.

[46] Bang YJ, Van Cutsem E, Fuchs CS (2019). KEYNOTE-585: Phase III study of perioperative chemotherapy with or without pembrolizumab for gastric cancer. Future Oncol.

[47] Shah MA, Bennouna J, Doi T (2021). KEYNOTE-975 study design: a Phase III study of definitive chemoradiotherapy plus pembrolizumab in patients with esophageal carcinoma. Future Oncol.

