# Supplementary material for: Perioperative chemotherapy with 5-FU, leucovorin, oxaliplatin, and docetaxel (FLOT) for esophagogastric adenocarcinoma: ten years real-life experience from a surgical perspective
Source: Langenbecks Arch Surg. 2023 Feb 10;408(1):81. doi: 10.1007/s00423-023-02822-7 (PMC9918580; doi:10.1007/s00423-023-02822-7)
Supplement: Supplementary file 1 — Supplementary file1 (DOCX 19 KB) [file 423_2023_2822_MOESM1_ESM.docx]

**Supplement table:** Summary of patients with distant metastasis in initial clinical staging (cM1)

| **cM1** | **FLOT (n=48)** | | **EPF (n=6)** | |
| --- | --- | --- | --- | --- |
| **cM category localizations** |  |  |  |  |
| hepatic | 7 | 14.6% | 1 | 16.7% |
| pulmonary | 2 | 4.2% | 2 | 33.3% |
| peritoneal | 16 | 33.3% | 1 | 16.7% |
| ovary | 1 | 2.1% | 1 | 16.7% |
| lymph nodes | 12 | 25.0% | 0 | 0% |
| adrenal gland | 3 | 6.3% | 0 | 0% |
| multiple sites | 7 | 14.6% | 1 | 16.7% |
| **cM category after preoperative chemotherapy** |  |  |  |  |
| **cM0** | 17 | 35.4% | 2 | 33.3% |
| **cM1** | 31 | 64.6% | 4 | 66.7% |
| hepatic | 5 | 16.3% | 1 | 25.0% |
| pulmonary | 1 | 3.2% | 0 | 0.0% |
| peritoneal | 10 | 32.3% | 1 | 25.0% |
| ovary | 1 | 3.2% | 1 | 25.0% |
| lymph nodes | 6 | 19.4% | 0 | 0.0% |
| adrenal gland | 3 | 9.7% | 0 | 0.0% |
| multiple sites | 5 | 16.1% | 1 | 25.0% |
| **cM category during surgery** |  |  |  |  |
| **cM0** | 23 | 47.9% | 3 | 50.0% |
| **cM1** | 25 | 52.1% | 3 | 50.0% |
| hepatic | 4 | 16.0% | 0 | 0.0% |
| peritoneal | 10 | 40.0% | 1 | 33.3% |
| ovary | 1 | 4.0% | 1 | 33.3% |
| lymph nodes | 5 | 20.0% | 0 | 0.0% |
| adrenal gland | 1 | 4.0% | 0 | 0.0% |
| multiple sites | 4 | 16.0% | 1 | 33.3% |
| **Resection of metastatic lesions** |  |  |  |  |
| **None** | 22 | 45.8% | 3 | 50.0% |
| **Yes** | 26 | 54.2% | 3 | 50.0% |
| hepatic | 4 | 15.4% | 0 | 0.0% |
| pulmonary | 1 | 3.8% | 0 | 0.0% |
| peritoneal | 10 | 38.5% | 1 | 33.3% |
| ovary | 1 | 3.8% | 1 | 33.3% |
| lymph nodes | 5 | 19.2% | 0 | 0.0% |
| adrenal gland | 1 | 3.8% | 0 | 0.0% |
| multiple sites | 4 | 15.4% | 1 | 33.3% |
| **(y)pM category** |  |  |  |  |
| **(y)pM0** | 24 | 50.0% | 3 | 50.0% |
| **(y)pM1** | 24 | 50.0% | 3 | 50.0% |
| hepatic | 4 | 16.7% | 0 | 0.0% |
| peritoneal | 10 | 41.7% | 1 | 33.3% |
| ovary | 1 | 4.2% | 1 | 33.3% |
| lymph nodes | 4 | 16.7% | 0 | 0.0% |
| adrenal gland | 1 | 4.2% | 0 | 0.0% |
| multiple sites | 4 | 16.7% | 1 | 33.3% |
